# Supplementary material for: Structural conservation of insulin/IGF signalling axis at the insulin receptors level in Drosophila and humans
Source: Nat Commun. 2023 Oct 7;14:6271. doi: 10.1038/s41467-023-41862-x (PMC10560217; doi:10.1038/s41467-023-41862-x)
Supplement: Supplementary file 3 — Reporting Summary [file 41467_2023_41862_MOESM3_ESM.pdf]

nature portfolio

Corresponding author(s): Andrzej M. Brzozowski

Last updated by author(s): 14/9/23

Reporting Summary

Nature Portfolio wishes to improve the reproducibility of the work that we publish. This form provides structure for consistency and transparency in reporting. For further information on Nature Portfolio policies, see our Editorial Policies and the Editorial Policy Checklist.

Statistics

For all statistical analyses, confirm that the following items are present in the figure legend, table legend, main text, or Methods section.

n/a	Confirmed	
☒	☐	The exact sample size (*n*) for each experimental group/condition, given as a discrete number and unit of measurement
☒	☐	A statement on whether measurements were taken from distinct samples or whether the same sample was measured repeatedly
☒	☐	The statistical test(s) used AND whether they are one- or two-sided<br>*Only common tests should be described solely by name; describe more complex techniques in the Methods section.*
☒	☐	A description of all covariates tested
☒	☐	A description of any assumptions or corrections, such as tests of normality and adjustment for multiple comparisons
☒	☐	A full description of the statistical parameters including central tendency (e.g. means) or other basic estimates (e.g. regression coefficient) AND variation (e.g. standard deviation) or associated estimates of uncertainty (e.g. confidence intervals)
☒	☐	For null hypothesis testing, the test statistic (e.g. *F*, *t*, *r*) with confidence intervals, effect sizes, degrees of freedom and *P* value noted<br>*Give P values as exact values whenever suitable.*
☒	☐	For Bayesian analysis, information on the choice of priors and Markov chain Monte Carlo settings
☒	☐	For hierarchical and complex designs, identification of the appropriate level for tests and full reporting of outcomes
☒	☐	Estimates of effect sizes (e.g. Cohen's *d*, Pearson's *r*), indicating how they were calculated

Our web collection on statistics for biologists contains articles on many of the points above.

Software and code

Policy information about availability of computer code

Data collection	cryoEM: EPU 2
Data analysis	SignalP 6.0 web service, RELION 3.1.2, Topaz 0.2.3 , Motioncorr2, CTFFIND-4.1, ChimeraX v 1.3, Modeller version 9.21, COOT v 0.9.8.7, Phenix, ISOLDE v 1.3.2, MolProbity, PyMOL, ClusPro server, CCP4MG, MicroCal PEAQ-ITC Analysis Software version 1.1.0.1262 , AlphaFold/2.0.0-foss-2020b

For manuscripts utilizing custom algorithms or software that are central to the research but not yet described in published literature, software must be made available to editors and reviewers. We strongly encourage code deposition in a community repository (e.g. GitHub). See the Nature Portfolio guidelines for submitting code & software for further information.

Data

Policy information about availability of data

All manuscripts must include a data availability statement. This statement should provide the following information, where applicable:

- Accession codes, unique identifiers, or web links for publicly available datasets
- A description of any restrictions on data availability
- For clinical datasets or third party data, please ensure that the statement adheres to our policy

The final models for DILP5:dmIR-ECD and cryo-EM reconstructions reported were deposited with the Electron Microscopy Data Base (EMD-16718) and the Protein Data Bank (PDB 8CLS). A Source Data File has been provided. Other raw data and biological material related to this study may be available directly from the authors

subject of collaborative agreements
.

Human research participants

Policy information about studies involving human research participants and Sex and Gender in Research.

Reporting on sex and gender	No human participants were involved in this research.
Population characteristics	N/A
Recruitment	N/A
Ethics oversight	N/A

Note that full information on the approval of the study protocol must also be provided in the manuscript.

Field-specific reporting

Please select the one below that is the best fit for your research. If you are not sure, read the appropriate sections before making your selection.

☒ Life sciences ☐ Behavioural & social sciences ☐ Ecological, evolutionary & environmental sciences

For a reference copy of the document with all sections, see nature.com/documents/nr-reporting-summary-flat.pdf

Life sciences study design

All studies must disclose on these points even when the disclosure is negative.

Sample size	CryoEM: a single representative grid (n - 1) proved sufficient to provide enough grid squares to generate representative set of particles. 4524 micrographs based on 4858 movies were used to extract the starting set of 1.2m particles.
Data exclusions	CryoEM: CryoEM particles were removed by careful exclusion of poorly resolved classes using the data processing pipeline described in the paper. In short, the initial autopicking using LoG resulted in 1.2m particles. Iterative reference-free 2D classifications removed noisy and contaminated classes yielding 134 visually selected good looking classes based on 319k particles, with simultaneous removal of damaged or imaging artifacts. These were truncated further to 30 good looking classes based on 72k particles with simultaneous. These 3D classed model and 1250 training particles were selected to train TOPAZ, to expand subsequently for 738k particles, providing ultimately good model for refinement based on 129k particles, as summarised in Supplementary Information Figure 3.
Replication	CryoEM; Replication and randomization are closely related procedures in cryo-EM structure determination. Cryo-EM single particle analysis intrinsically involves averaging many independent observations.
Randomization	CryoEM: The gold standard Fourier shell correlation curve that is calculated during refinement is done by randomly splitting the data into two halves and refining each half individually. Resolution was assessed by the Fourier shell correlation between the half-maps at the 0.143 threshold criteria, indicating the resolution which is reproducible from this data set.
Blinding	CryoEM: Blinding was not employed since it is not common or applicable to the methods described in this study.

Reporting for specific materials, systems and methods

We require information from authors about some types of materials, experimental systems and methods used in many studies. Here, indicate whether each material, system or method listed is relevant to your study. If you are not sure if a list item applies to your research, read the appropriate section before selecting a response.

Materials & experimental systems

n/a	Involved in the study
☒	☐ Antibodies
☐	☒ Eukaryotic cell lines
☒	☐ Palaeontology and archaeology
☒	☐ Animals and other organisms
☒	☐ Clinical data
☒	☐ Dual use research of concern

Methods

n/a	Involved in the study
☒	☐ ChIP-seq
☒	☐ Flow cytometry
☒	☐ MRI-based neuroimaging

Eukaryotic cell lines

Policy information about cell lines and Sex and Gender in Research

Cell line source(s)	Sf9 cells: ThermoFisher Catalog number: 11496015
Authentication	No cell authentication was performed as standard Sf9 cells were used.
Mycoplasma contamination	Cell lines were not tested for mycoplasma contamination.
Commonly misidentified lines (See ICLAC register)	No commonly misidentified lines were used.
